# Reply to: Comment on “Inferring broken detailed balance in the absence of observable currents”

**DOI:** 10.1038/s41467-024-52603-z

**Published:** 2024-10-07

**Authors:** Gili Bisker, Ignacio A. Martínez, Jordan M. Horowitz, Juan M. R. Parrondo

**Affiliations:** 1 Department of Biomedical Engineering, Faculty of Engineering, Tel Aviv University, Tel Aviv, Israel; 2 Center for Physics and Chemistry of Living Systems, Center for Nanoscience and Nanotechnology, Center for Light-Matter Interaction, Tel Aviv University, Tel Aviv, Israel; 3 Electronics and Information Systems, Ghent University, Technologiepark Zwijnaarde 15, Gent, Belgium; 4 Department of Biophysics, University of Michigan, Ann Arbor, MI USA; 5 Center for the Study of Complex Systems, University of Michigan, Ann Arbor, MI USA; 6 Department of Physics, University of Michigan, Ann Arbor, MI USA; 7 Departamento de Estructura de la Materia, Física Termica y Electronica and GISC, Universidad Complutense de Madrid, Madrid, Spain

**Keywords:** Complex networks, Statistical physics

**replying to** D. Hartich & A. Godec *Nature Communications* 10.1038/s41467-024-52602-0 (2024)

In^1^, Hartich and Godec (HG) present a counterexample that apparently refutes our results in^2^. While the content of their Comment is essentially correct, it does not invalidate our results but rather raises an interesting question on the effect of coarse-graining on irreversibility, which we discuss in detail below. However, the way they write the Comment is slightly misleading. They highlight the fact that we never tested Eq. (4) in ref. ^2^, implicitly suggesting that this equation is wrong. Eqs. (2–4) are an exact expression of the Kullbak-Leibler divergence (KLD) between a semi-Markov chain and its time reverse. There is no need of a test of Eq. (4) since it is an exact result, whose rigorous mathematical proof is given in the section “*Methods*” of ref. ^2^. Moreover, HG claim that our results and those of Wang and Qian^3^ are “diametrically opposing”. We want to stress that this is not true. Both our paper and^3^ are fully correct and perfectly compatible.

The example in^1^ does not compromise the validity of Eq. (4). Instead, it calls into question Eq. (1) in our paper^2^, which asserts that the KLD between an observed trajectory and its time reversal is a lower bound to the physical entropy production. This claim is widely used in the literature and based on a well-known property of the KLD. The KLD between two stochastic processes measures our capacity to distinguish between them using data. If we remove information about these processes, by adding noise to the data or decimating the underlying network, it should be clear that this capacity decreases and, consequently, the KLD decreases. Since the entropy production is equal to the KLD between the microscopic trajectory and its time reversal^4–6^, the KLD between a coarse-grained trajectory and its time reversal is a lower bound on the actual entropy production.

In more precise mathematical terms, this argument is based on the following property of the KLD between two distributions *p*_*X*_ and *q*_*X*_ of a random variable *X*:

1 $$D({p}_{X}| | {q}_{X})\ge D\left({p}_{f(X)}| | {q}_{f(X)}\right)$$

where *f*(*x*) is an arbitrary (possibly random) function. The equality holds if *f* is one-to-one and deterministic. The function *f* can represent a coarse-graining when several states {*x*_1_, …, *x*_*m*_} coalesce into a single state *f*(*x*_1_) = ⋯ = *f*(*x*_*m*_), or the removal of the information associated to a particular variable, when for instance *x* = (*x*^(1)^, *x*^(2)^) and *f*(*x*^(1)^, *x*^(2)^) = *x*^(1)^.

The inequality (1) has a direct and easy interpretation: by manipulating the available data, that is, by transforming the data according to the function *f*, one cannot increase the distinguishability between the two probability distributions, *p* and *q*.

If *X* is a microscopic trajectory and *ΘX* is its time reversal, the entropy production Δ*S* verifies^4,5^

2 $$\Delta S=kD({p}_{X}| | {p}_{\Theta X}).$$

where *k* is Boltzmann constant. If we apply Eq. (1) to this expression, we obtain

3 $$\Delta S\ge kD\left({p}_{f(X)}| | {p}_{f(\Theta X)}\right).$$

However, Eq. (1) in our work^2^ slightly differs from this inequality. There, we assume that the observer has access to a coarse-grained trajectory *f*(*X*) and constructs the time reversal as *Θf*(*X*). Hence, to have

4 $$\Delta S\ge kD\left({p}_{f(X)}| | {p}_{\Theta f(X)}\right)$$

from Eq. (3), a sufficient condition is that coarse-graining and time reversal commute, *f*(*ΘX*) = *Θf*(*X*).

The example in^1^ does not fulfill this commutation relation, as HG mention in their comment. In Fig. 1, we introduce a simplified version of the counterexample discussed in^1^ that illustrates the origin of this issue. The example is a kinetic network at equilibrium with four states, *A*, *B*, *C*, and *b*. Suppose that the observer does not have access to state *b*. There are different options for constructing the coarse-grained trajectory. The one discussed by HG consists of taking the arrivals at the states *A*, *B*, and *C* as jumps between states in the coarse-grained trajectory. In Fig. 1 (*bottom*), we plot a sketch of a reversible microscopic trajectory (thin black lines) *X* and the corresponding coarse-grained trajectory using this prescription (red dashed lines). Since *X* is reversible, *X* = *ΘX*, and the resulting trajectory is irreversible, hence *Θf*(*X*) ≠ *f*(*X*) = *f*(*ΘX*). The irreversibility of the red trajectory is revealed by the waiting time distributions: in the forward trajectory, *f*(*X*), the system takes two units of time to jump down from *B* to *C* and one unit of time to jump up from *B* to *A*, whereas these waiting times are swapped when the trajectory is reversed. Notice that this decimation procedure is nonlocal in time, i.e., the state of the decimated trajectory at time *t* is not a function of the micro-state at time *t* (open circles), but depends on the past.

**Fig. 1:**
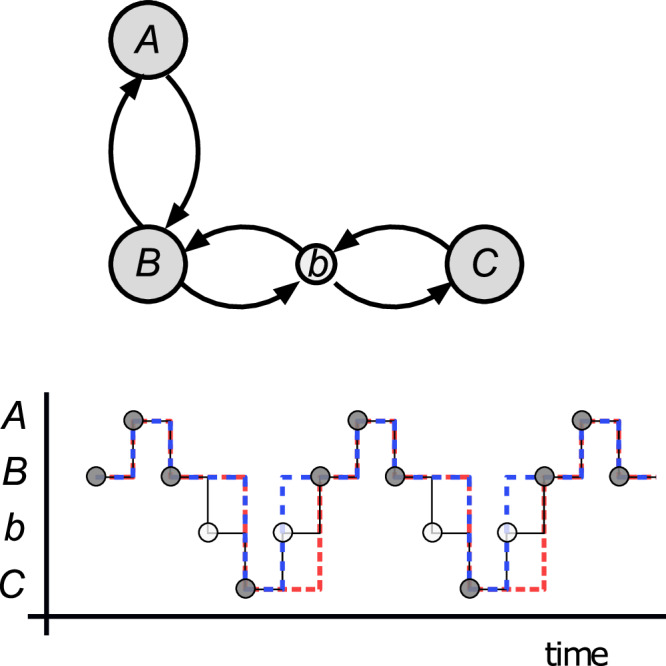
Local versus non-local decimation. *Top)* A simplified version of the counterexample introduced in^1^. We decimate state *b*. *Bottom)* We plot microscopic trajectory (thin black lines) and the trajectories (dashed lines) resulting from different decimation procedures, where state *b* (open circles) is not observable. The blue dashed line is the result of lumping states *B* and *b*. Since the decimation is local in time, decimation, and time reversal commute, *f*(*ΘX*) = *Θf*(*X*). Here, the microscopic trajectory *X* is reversible, hence *ΘX* = *X*, and the commutation implies that *f*(*X*) is also reversible, as shown in the figure. On the other hand, the red dashed line results from the decimation procedure considered in^1^, which is based on the arrivals at states *A* and *B*. This decimation does not commute with the time-reversal operation, and the resulting trajectory is irreversible *Θf*(*X*) ≠ *f*(*X*) = *f*(*ΘX*): in the red trajectory the system takes two units of time to jump down from *B* to *C* and one unit of time to jump up from *B* to *A*; whereas these waiting times are swapped when the trajectory is reversed. Notice that this decimation procedure is non-local in time, i.e., the state of the decimated trajectory at time *t* is not a function of the micro-state at time *t* (open circles), but depends on the past.

We also plot in Fig. 1 (*bottom*), the trajectory resulting from lumping states *B* and *b* (blue dashed lines), which is reversible. This procedure is local in time since the hidden state *b* is always mapped onto *B*, and consequently decimation and time reversal commute, *f*(*ΘX*) = *Θf*(*X*).

Summarizing, HG comment is interesting since it points out that a sufficient condition for Eq. (1) in ref. ^2^ to be valid is that coarse-graining and time reversal commute. Consequently, our claim in^2^ that waiting time distributions unravel “irreversibility in hidden degrees of freedom arising in any time-series measurement of an arbitrary experimental setup”, should be limited to experimental setups where the condition is fulfilled. The violation of this condition was not considered in our paper because we implicitly assumed coarse-graining procedures that are local in time and consequently commute with the time-reversal operation, as implied in the description of the decimation process in the main text and in the “*Methods*” section in^2^.

Finally, another possible criticism of our paper^2^ follows from the following fact: If the decimation of a Markov chain yields a 1^*st*^ order semi-Markov process, then the KLD between waiting time distributions vanishes if the KLD between affinities is zero, i.e., ${\dot{S}}_{{{{\rm{aff}}}}}=0$ implies ${\dot{S}}_{{{{\rm{WTD}}}}}=0$ (these KLD’s are introduced in Eqs. (3) and (4) of^2^). This means that, in these cases, the KLD between waiting time distributions cannot detect entropy production in absence of currents. Hence, the claim we made in the title of our paper^2^ is only valid if the decimation yields a 2^*nd*^ order semi-Markov process.

One could then argue that the paper would be clearer if we remove Eq. (4) from ref. ^2^, which is valid for 1^*st*^ order semi-Markov processes, and derive directly Eq. (6), which is valid for 2^*nd*^ order semi-Markov processes. This remark refers to the clarity of the presentation, which is a subjective matter, and not to the validity of Eq. (4) and (6) in^2^, which are both exact and correct. However, we still have reasons to consider Eq. (4) useful and informative. First, 2^*nd*^ order semi-Markov processes can be reduced to 1^*st*^ order by considering the evolution of two consecutive states. Actually, we use this reduction and Eq. (4) to derive Eq. (6). Second, Eq. (4) is a relevant result by itself. Up to our knowledge, Eqs. (2-4) are the first exact expression for the KLD between a 1^*st*^ order semi-Markov process and its time reversal. Third, Eq. (6) is more complicated than Eq. (4) because it involves three-step jumps. Eq. (4) is simpler, more fundamental, and provides a more intuitive picture of how waiting time distributions contribute to the KLD.

## Data Availability

We do not analyze or generate any datasets since our work proceeds within a theoretical and mathematical approach.

## References

[1] Hartich, D. & Godec, A. Comment on “Inferring broken detailed balance in the absence of observable currents”. *Nat. Commun*. 10.1038/s41467-024-52602-0 (2024). 10.1038/s41467-024-52602-0 PMC11458581 39375350

[2] Martínez, I. A., Bisker, G., Horowitz, J. M. & Parrondo, J. M. R. Inferring broken detailed balance in the absence of observable currents. *Nat. Commun.* **10**, 3542 (2019). 31387988 10.1038/s41467-019-11051-w PMC6684597

[3] Wang, H. & Qian, H. On detailed balance and reversibility of semi-Markov processes and single-molecule enzyme kinetics. *J. Math. Phys.* **48**, 013303 (2007).

[4] Kawai, R., Parrondo, J. M. R. & den Broeck, C. V. Dissipation: The Phase-Space Perspective. *Phys. Rev. Lett.* **98**, 080602 (2007). 17359081 10.1103/PhysRevLett.98.080602

[5] Roldán, E. & Parrondo, J. M. R. Estimating Dissipation from Single Stationary Trajectories. *Phys. Rev. Lett.* **105**, 150607 (2010). 21230886 10.1103/PhysRevLett.105.150607

[6] Roldán, É., Barral, J., Martin, P., Parrondo, J. M. R. & Jülicher, F. Quantifying entropy production in active fluctuations of the hair-cell bundle from time irreversibility and uncertainty relations. *N. J. Phys.* **23**, 083013 (2021).

